# Body mass index and smoking-related lung cancer risk in the Singapore Chinese Health Study

**DOI:** 10.1038/sj.bjc.6605496

**Published:** 2009-12-15

**Authors:** W-P Koh, J-M Yuan, R Wang, H-P Lee, M C Yu

**Affiliations:** 1 Department of Epidemiology and Public Health, Yong Loo Lin School of Medicine, National University of Singapore, Singapore, Singapore; 2 The Masonic Cancer Center, University of Minnesota, Minneapolis, MN, USA; 3 Division of Epidemiology and Community Health, School of Public Health, University of Minnesota, Minneapolis, MN, USA

**Keywords:** body mass index, smoking, lung cancer, prospective cohort, Chinese, Singapore

## Abstract

**Background:**

Smokers with low body mass index (BMI) may be more susceptible to lung cancer.

**Methods:**

We prospectively examined the association between baseline BMI and lung cancer risk in the Singapore Chinese Health Study, a cohort of 63 257 Chinese enrolled between 1993 and 1998.

**Results:**

After adjustment for smoking intensity and duration, BMI was inversely associated with risk of lung cancer among current smokers (*P* for trend=0.0004). Current smokers at different dosage of smoking with low BMI had significantly higher risk for lung cancer than those with high BMI. Hazard ratios (95% confidence intervals) of lung cancer for heavy smokers with BMI of ⩾28, 24–<28, 20–<24, and <20 kg m^−2^ were 6.37 (2.10–19.30), 9.01 (5.04–16.10), 8.53 (6.35–11.5), and 11.12 (6.60–18.70), respectively, as compared with nonsmokers. BMI had no modifying effects on lung cancer risk among nonsmokers and former smokers.

**Conclusion:**

Smokers with lower BMI may experience an enhanced risk of lung cancer. The findings have significant public-health implication given the increase in smoking prevalence in developing countries, where people still have relatively low BMI.

Lung cancer has the highest incidence and mortality rates in many countries, and cigarette smoking is estimated to account for as high as 90% of all cases (IARC, 1986). However, there is a wide variation in lung cancer incidence among smokers, as it has been estimated that approximately only 16% of current smokers among men and 10% of current smokers among women would die from lung cancer by 75-years of age (Peto *et al*, 2000). The determinants of risk include duration and intensity of smoking, exposure to asbestos (O’Reilly *et al*, 2007), and radiation (Jacob *et al*, 2007); other factors include uptake and metabolism of tobacco carcinogens (Yuan *et al*, 2009), variations in nicotine receptor-related genes (Amos *et al*, 2008; Hung *et al*, 2008), inflammatory response to the tobacco-induced lung damage (Engels *et al*, 2007) and DNA repair (Hu *et al*, 2005).

Although certain studies (mainly in western populations) have shown an inverse association between body mass index (BMI) and lung cancer risk (Knekt *et al*, 1991; Kabat and Wynder, 1992; Kark *et al*, 1995), this association is often criticised as caused by inadequate adjustment for cigarette smoking, as both smoking status and its intensity are strongly related to both BMI and lung cancer incidence. In fact, this inverse association was largely eliminated when the analysis was limited to nonsmokers (Henley *et al*, 2002). Recently, a prospective study of BMI and lung cancer mortality among 220 000 Chinese men, a relatively lean population, found that low BMI was associated with increased risk only among current smokers (Yang *et al*, 2009). In this study, we prospectively examined this subject in a cohort of 63 257 middle-aged and older Chinese men and women in Singapore, a population with a mean BMI of 23 kg m^−2^. We also examined whether BMI has a modifying effect on smoking- and nonsmoking-related lung cancer risk.

## Materials and methods

The design of the Singapore Chinese Health Study has been described previously (Hankin *et al*, 2001). Briefly, the cohort was drawn from permanent residents or citizens of Singapore, who resided in government-built housing estates. The age eligibility criterion was 45–74 years and recruitment was restricted to the two major dialect groups of Chinese in Singapore, the Hokkiens and the Cantonese. Between April 1993 and December 1998, 63 257 subjects (about 85% of eligible subjects approached) were recruited. In this study, we excluded 1936 individuals with a baseline history of invasive cancer (except non-melanoma skin cancer) or superficial, papillary bladder cancer from the analysis, leaving 61 321 subjects. The study was approved by the Institutional Review Boards of the National University of Singapore and the University of Minnesota.

At the time of recruitment, an in-person interview was conducted at the subject's residence by a trained interviewer using a structured questionnaire. This covered demographics, lifetime use of tobacco (cigarettes and water-pipe), current physical activity, menstrual/reproductive history (women only), occupational exposure, medical history, and family history of cancer. Current diet was assessed using a 165-item food frequency questionnaire that has been validated against a series of 24-h dietary recall interviews (Hankin *et al*, 2001) and selected biomarker studies (Seow *et al*, 1998a, 1998b) conducted on random subsets of cohort participants.

The subjects were enquired for their current weight and height by the interviewer. BMI was calculated as the current weight in kilograms divided by height in metres squared. There were 9781 cohort participants with unknown weight, 97 with unknown height, and 192 with both unknown weight and height. Their BMIs were calculated using imputed weight and/or height derived from the linear regression equation: 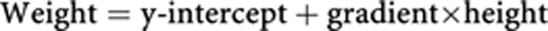 where values for the y-intercept and gradient were obtained from gender-specific weight-height regression lines drawn from all cohort participants with known heights and weights. If only weight or height was missing, the linear regression equation was used to estimate the missing value. If both weight and height were missing, the missing height was assigned the sex-specific median value and missing weight value calculated from the linear regression equation. Subjects were classified into never smoker, former smoker, and current smoker as described previously (Koh *et al*, 2005).

Deaths and incident cancers among cohort members were identified by record linkage of the cohort with databases from the population-based Singapore Cancer Registry and the Singapore Registry of Births and Deaths. The nationwide cancer registry, in place since 1968, has been shown to be comprehensive in its ascertainment of cancers (Parkin *et al*, 2002). As of April 2008, only 27 cases were lost to follow-up because of migration out of Singapore. As of 31 December 2006, 1042 cohort participants who were cancer-free at baseline had developed lung cancer. Of these, 912 (87.5%) were diagnosed histologically and they were confirmed via manual review of pathology reports by a medically trained research staff; 96 (9.2%) cases were diagnosed clinically and 34 (3.3%) cases were identified through death certificates.

### Data analysis

The subjects were classified into groups of BMI<20, 20–<24, 24–<28, and ⩾28 kg m^−2^, revised categories that are more appropriate to characterise obesity in Asian populations. For each subject, person-years of follow-up were counted from the date of recruitment to the date of diagnosis of lung cancer, death, or 31 December 2006, whichever occurred first. Proportional hazards (Cox) regression methods were used to examine the associations with BMI and other exposure factors (Cox, 1972). The strength of the association was measured by hazard ratio (HR) and its 95% confidence interval (CI) and *P*-value. All Cox's regression models included age at recruitment (years), gender, dialect group (Hokkien, Cantonese), and year of recruitment. When examining the BMI–lung cancer association, the models also included level of education (no formal education, primary school, secondary or higher education), number of cigarettes smoked per day (never smoker, 1–12, 13–22, or 23+), number of years of smoking (never smokers, 1–19, 20–39, or 40+), number of years since quitting smoking (continuous smokers, <1, 1–4, 5–19, 20+, or never smokers), and dietary intakes of *β*-cryptoxanthin (*μ*g 1000 kcal^−1^), factors that were associated with lung cancer risk in the study population (Yuan *et al*, 2003). Analyses were carried out for men and women separately, as well as combined for both sexes. As all exposure–lung cancer risk associations were comparable between men and women, results were presented for both sexes combined with adjustment for gender. Statistical computing was conducted using SAS version 9.1 (SAS Institute, Cary, NC, USA) statistical software package. All *P*-values quoted are two sided.

## Results

As of 31 December 2006, 1042 cohort participants (717 males and 325 females) were diagnosed with lung cancer, at a mean age (±s.d.) of 69.3 (±7.2) years for men and 69.6 (±8.4) years for women. The mean time interval between baseline interview and diagnosis was 10.7 years (range, from 1 month to 13.9 years). Smoking was a strong risk predictor: compared with never smokers, current smokers had an HR of 5.85 (95% CI=4.99–6.87) for lung cancer. The risk increased monotonically with increasing number of cigarettes per day and number of years of smoking. Among former smokers, number of years since quitting smoking habit was associated with monotonically reduced risk of lung cancer (Table 1).

The mean BMI (±s.d.) is 23.0 (±3.2) kg m^−2^ in men and 23.2 (±3.3) kg m^−2^ in women. After adjustment for smoking history (number of cigarettes smoked per day, number of years of smoking, and number of years since quitting smoking for former smokers) and other potential confounders, low BMI was associated with a statistically significant increased risk of lung cancer (*P* for trend=0.0004). We also carried out parallel analysis after excluding subjects with imputed BMI (10 070 subjects, 221 lung cancers), the results being essentially identical with those based on the entire cohort (Table 2). This inverse BMI-lung cancer association was confined to current smokers. Current smokers with BMI <20 kg m^−2^ had an HR of 1.91 (95% CI=1.12–3.25) for lung cancer compared with current smokers with comparable smoking history but with BMI ⩾28 kg m^−2^ (Table 3); after excluding current smokers with imputed BMI (2280 subjects, 144 lung cancers), the corresponding HR was 1.92 (95% CI=1.13–3.28).

There was no evidence that the inverse BMI–lung cancer association was influenced significantly by the duration of follow-up. Exclusion of lung cancer cases and person-years of observation in the first 5 years post-enrollment did not materially alter the inverse association between BMI and lung cancer risk. Similarly, exclusion of subjects with pre-existing respiratory diseases (tuberculosis, allergic rhinitis, rhinitis, sinusitis, and asthma) and other diseases/conditions (coronary heart disease, stroke, diabetes, peptic ulcer, partial removal of stomach, and polyps of intestines) also did not materially change the inverse association.

We further examined the interactive effects of BMI and cigarette smoking on risk. Among subjects with BMI ⩾28 kg m^−2^, current smokers had an HR of 3.21 (95% CI=1.58–6.51) compared with nonsmokers; the corresponding figures for BMI <20 kg m^−2^ were 7.21 (95% CI=4.84–10.75) (Table 4). Similarly, HRs (95% CIs) for heavy smokers (23+ cigarettes per day) relative to nonsmokers within each category of BMI ⩾28, 24–<28, 20–<24, and <20 kg m^−2^ were 6.37 (2.10–19.30), 9.01 (5.04–16.10), 8.53 (6.35–11.5), and 11.12 (6.60–18.70), respectively (Table 4).

## Discussion

Our results showed an inverse, dose–dependent association between BMI and lung cancer risk among current smokers of both sexes, after adjustment for duration (years of smoking) and intensity (number of cigarettes smoked per day) of smoking. This association remained after exclusion of subjects with pre-existing disease or the first 5-years of follow-up data (both person-years and lung cancer cases). There was no association between BMI and lung cancer risk in either former or never smokers.

This study shows that the inverse BMI–lung cancer risk association was consistent across a range of smoking intensity. Hence, for the same dose of smoking, smokers with low BMI (<20 kg m^−2^) had a significantly elevated risk of lung cancer relative to smokers with high BMI. Thus, lung cancers in people with low BMI could be from factors associated with leanness or the biological effect of leanness itself. It is biologically plausible that leanness may modulate the carcinogenic effects of smoking directly. Cross sectional and longitudinal studies have shown that both BMI itself and the decrease in BMI inversely correlated with the level of urinary 8-hydroxydeoxyguanosine (8-OHdG), a marker of oxidative DNA damage in smokers, suggesting that BMI may serve as an independent marker for host susceptibility to smoking-related cancer (Loft *et al*, 1992; Mizoue *et al*, 2006, 2007). Smoking may induce weight loss by increasing the metabolic rate, which also leads to higher production of cellular-reactive species, a carcinogenic pathway that is, therefore, enhanced among lean smokers (Loft *et al*, 1992).

In a 16-year prospective study of a Chinese cohort recruited during 1970s, the relative risk of lung cancer in male smokers was 3.8 (Chen *et al*, 1997) compared with those typically 20-fold reported in western populations (Peto *et al*, 1994). This apparently lower risk associated with smoking among Chinese has been attributed to lighter dose of smoking and the relatively late age of starting to smoke in China (Chen *et al*, 1997). There are disturbing observations that the mean cigarette consumption by Chinese men is increasing rapidly (Liu *et al*, 1998) and that smoking is starting at an earlier age (Yang, 1997). Lung cancer mortality attributed to smoking in China can therefore be expected to rapidly increase from the current 63% (Chen *et al*, 1997) to the above 90% levels noted in certain industrialised countries (Ezzati and Lopez, 2003). Our study suggests that for the same intensity of smoking, the risk of lung cancer may be higher among the leaner Chinese than their western counterparts with higher BMI. Projected lung cancer estimates based on relative risks derived from western populations with heavier BMI may therefore seriously underestimate the true burden among the leaner Chinese.

The strengths of our study included (1) the population-based prospective study design with a large number of genetically homogeneous subjects, (2) detailed information on smoking and potential confounders including diet and other lifestyle factors, and medical history, (3) virtually complete follow-up of deaths and incident cancers, and (4) histological confirmation of most lung cancers (87.5%). A potential limitation was the use of self-reported heights and weights at baseline, but these have consistently been shown to be reliable with relatively small mean errors (Gorber *et al*, 2007). In addition, although the risk estimates were not significantly different between the two genders, the relatively small number of current smokers in women prevented our detecting a significant gender difference in the relationship between BMI and lung cancer risk among current smokers.

Given the strong impact of cigarette smoking on both BMI and lung cancer risk, inadequate adjustment for smoking may produce a spurious association with BMI (Kabat and Wynder, 1992; Kanashiki *et al*, 2005). To adjust for smoking, we included details of numbers of cigarettes smoked and years of smoking at baseline interview in multivariate regression models. Studies with short follow-up may also be confounded by preclinical weight loss or by pre-existing disease, which may contribute to both leanness and lung cancer risk. The exclusion of pre-existing clinical disease or subjects with <5 years of follow-up did not materially change our results.

Prospective studies have largely reported null associations between BMI and lung cancer risk among never and former smokers either among women (Kabat *et al*, 2008) or both genders in various population (Henley *et al*, 2002; Inoue *et al*, 2004; Kanashiki *et al*, 2005), and recently in large prospective Asian cohorts (Jee *et al*, 2008; Yang *et al*, 2009). Our data are consistent with these previous studies.

The finding of an inverse association between BMI and lung cancer risk in current smokers has important implications given the marked increase in smoking prevalence in China and other developing countries, where people still have relatively low body weights.

## Figures and Tables

**Table 1:** Cigarette smoking in relation to lung cancer risk, the Singapore Chinese Health Study 1993–2006

**Cigarette smoking**	**No. of cases**	**Rate^a^**	**HR (95% CI)^b^**
*Smoking status*			
Never smokers	287	68	1.00
Former smokers	156	200	2.24 (1.81–2.78)
Current smokers	599	387	5.85 (4.99–6.87)
			
*No. of cigarettes per day*			
Never smokers	287	65	1.00
1–12	193	281	4.32 (3.55–5.23)
13–22	259	390	6.61 (5.46–8.02)
23+	147	670	9.49 7.58–11.88)
*P* for trend			<0.0001
			
*No. of years of smoking*			
Never smokers	287	68	1.00
1–19	30	109	1.52 (1.03–2.24)
20–39	213	210	3.20 (2.63–3.89)
40+	512	442	7.11 (5.98–8.45)
*P* for trend			<0.0001
			
*No. of years since quitting smoking*			
Current smokers	599	387	1.00
<1	24	480	0.91 (0.60–1.37)
1–4	40	284	0.51 (0.37–0.71)
5–19	66	194	0.37 (0.28–0.47)
20+	26	106	0.21 (0.14–0.31)
*P* for trend			<0.0001

a Rate per 100 000 person-years adjusted for age and sex.

b Hazard ratios (HRs) were adjusted for age at baseline, sex, dialect group and year of interview; confidence interval (CI).

**Table 2:** Body mass index (BMI) in relation to lung cancer risk, the Singapore Chinese Health Study 1993–2006

	**Whole cohort**			**Excluding subjects with imputed BMI**		
**Body mass index (kg m^−2^)**	**No. of cases**	**Rate^a^**	**HR (95% CI)^b^**	**No. of cases**	**Rate^a^**	**HR (95% CI)^b^**
⩾28	49	120	1.00	49	116	1.00
24–<28	164	111	0.91 (0.66–1.25)	164	110	0.92 (0.67–1.27)
20–<24	609	167	1.18 (0.88–1.59)	388	155	1.22 (0.91–1.65)
<20	220	218	1.34 (0.98–1.83)	220	213	1.37 (1.00–1.88)
*P* for trend			0.0004			0.0002

a Rate per 100 000 person-years adjusted for age and sex.

b Hazard ratios (HRs) were adjusted for age at baseline, sex, dialect group and year of interview, level of education, number of cigarettes smoked day, number of years of smoking, number of years since quitting smoking for former smokers, and dietary intake of *β*-cryptoxanthin; confidence interval (CI).

**Table 3:** Body mass index (BMI) in relation to lung cancer risk by smoking status, the Singapore Chinese Health Study 1993–2006

	**Never smokers**			**Former smokers**			**Current smokers**		
**BMI (kg m^−2^)**	**Cases**	**Rate^a^**	**HR (95% CI)^b^**	**Cases**	**Rate^a^**	**HR (95% CI)^c^**	**Cases**	**Rate^a^**	**HR (95% CI)^c^**
⩾28	23	78	1.00	11	152	1.00	15	217	1.00
24–<28	50	52	0.69 (0.42–1.13)	40	217	0.98 (0.50–1.91)	74	292	1.15 (0.66–2.01)
20–<24	176	75	1.01 (0.65–1.56)	82	208	0.85 (0.45–1.60)	351	391	1.58 (0.94–2.65)
<20	38	60	0.93 (0.55–1.56)	23	202	0.85 (0.41–1.75)	159	503	1.91 (1.12–3.25)
*P* for trend			0.31			0.46			<0.0001

a Rate per 100 000 person-years adjusted for age and sex.

b Hazard ratios (HRs) were adjusted for age at baseline, sex, dialect group and year of interview, level of education, and dietary intake of *β*-cryptoxanthin; confidence interval (CI).

c Further adjusted for number of cigarettes smoked day, number of years of smoking, and number of years since quitting smoking (for former smokers only).

**Table 4:** Cigarette smoking in relation to lung cancer risk by levels of body mass index, the Singapore Chinese Health Study 1993–2006

	**⩾28 kg m^−2^**		**24–<28 kg m^−2^**		**20–<24 kg m^−2^**		**<20 kg m^−2^**	
	**Cases**	**HR (95% CI)^a^**	**Cases**	**HR (95% CI)^a^**	**Cases**	**HR (95% CI)^a^**	**Cases**	**HR (95% CI)^a^**
*Smoking status*								
Never smokers	23	1.00	50	1.00	176	1.00	38	1.00
Former smokers	11	1.99 (0.88–4.52)	40	2.96 (1.84–4.76)	82	1.97 (1.48–2.62)	23	2.46 (1.40–4.32)
Current smokers	15	3.21 (1.58–6.51)	74	5.50 (3.64–8.32)	351	5.20 (4.22–6.41)	159	7.21 (4.84–10.75)
								
*No. of cigarettes per day* b								
1–12	6	2.90 (1.15–7.27)	21	3.65 (2.12–6.28)	105	3.65 (2.82–4.73)	61	6.18 (3.98–9.58)
13–22	4	2.21 (0.71–6.86)	31	5.41 (3.23–9.05)	161	6.39 (4.98–8.20)	63	7.92 (5.01–12.53)
23+	5	6.37 (2.10–19.3)	22	9.01 (5.04–16.1)	85	8.53 (6.35–11.5)	35	11.12 (6.60–18.7)
*P* for trend		0.001		<0.0001		<0.0001		<0.0001

a Hazard ratios (HRs) were adjusted for age at baseline, sex, dialect group and year of interview, level of education, and dietary intake of *β*-cryptoxanthin; confidence interval (CI).

b Among current smokers only; all HRs were relative to never smokers.
